# Transforming acidic coiled-coil protein-3: a novel marker for differential diagnosis and prognosis prediction in endocervical adenocarcinoma

**DOI:** 10.1186/s10020-021-00298-z

**Published:** 2021-06-10

**Authors:** Yan-Lin Wen, Shu-Mei Yan, Wei Wei, Xia Yang, Shi-Wen Zhang, Jing-Ping Yun, Li-Li Liu, Rong-Zhen Luo

**Affiliations:** 1 Sun Yat-Sen University Cancer Center, State Key Laboratory of Oncology in South China, Collaborative Innovation Center for Cancer Medicine, Guangzhou, 510060 China; 2 Department of Pathology, Sun Yat-Sen University Cancer Center, 651# Dong Feng Road East, Guangzhou, 510060 Guangdong China; 3 Department of Gynecological Oncology, Sun Yat-Sen University Cancer Center, Guangzhou, 510060 China

**Keywords:** TACC3, ECA, HPVA, NHPVA, Prognosis

## Abstract

**Background:**

Endocervical adenocarcinoma (ECA) is further classified as human papillomavirus (HPV)-associated (HPVA) or non-HPVA (NHPVA), per the International Endocervical Adenocarcinoma Criteria and Classification (IECC). HPVA is a glandular tumor with stromal invasion and/or exophytic expansile-type invasion, associated with the typical molecular characteristics of high-risk HPV (HR-HPV) infection. Transforming acidic coiled-coil protein-3 (TACC3),an oncogene that is frequently abnormally expressed,represents a vital biomarker for multiple human malignancies. This study aimed to examine the role of TACC3 in the diagnosis and prognosis of ECA.

**Methods:**

We analyzed 264 patients with ECA who underwent surgical resection, classifying their tumors into HPVA and NHPVA subtypes. The expression levels of TACC3, P16, MLH1, PMS2, MSH2, MSH6 and Ki-67 in tumors were evaluated by tissue microarray using immunohistochemistry (IHC). HPV subtypes were identified in formalin-fixed paraffin-embedded (FFPE) ECA tissues by the polymerase chain reaction (PCR).

**Results:**

ECA samples showed increased TACC3 expression relative to adjacent non-carcinoma samples. TACC3 expression was higher in HPVA than in NHPA. In the HPVA subtype, high TACC3 expression was significantly correlated with P16-positive, Ki-67-high expression. Furthermore, TACC3 levels were significantly related to tumor histological type (*P* = 0.006), nerve invasion (*P* = 0.003), differentiation (*P* = 0.004), surgical margin (*P* = 0.012), parametrium invasion (*P* = 0.040), P16 expression (*P* < 0.001), and Ki-67 (*P* = 0.004). Additionally, Kaplan–Meier analysis showed that TACC3 upregulation was associated with poor overall survival (OS, *P* = 0.001), disease-free survival (DFS, *P* < 0.001), and recurrence survival (*P* < 0.001). Multivariate analysis indicated that elevated TACC3 expression served as a marker to independently predict ECA prognosis. ROC curve analyses indicated that TACC3, P16, and HPV subtypes showed similar utility for distinguishing HPVA from NHPVA, with areas under the ROC curves of 0.640, 0.649, and 0.675, respectively. The combination of TACC3 and HPV subtypes improved the diagnostic performance of ECA compared with TACC3, P16, and HPV subtypes alone.

**Conclusions:**

Taken together, our findings identify that TACC3 is a promising complementary biomarker for diagnosis and prognosis for patients with ECA.

**Supplementary Information:**

The online version contains supplementary material available at 10.1186/s10020-021-00298-z.

## Introduction

Cervical adenocarcinoma accounts for 15-20% of cervical cancer, and the incidence rate and incidence rate are increasing (Ward 2012). According to the International Endocervical Adenocarcinoma Criteria and Classification (IECC), endocervical adenocarcinoma (ECA) can be divided into HPV-associated adenocarcinoma (HPVA) or non-HPV-associated adenocarcinoma (NHPVA), based on morphological characteristics associated with human papillomavirus (HPV) infection (Stolnicu 2019).Apical mitoses and apoptotic bodies are easily recognized in the HPVA subtype (Hodgson 2019). HPVA tends to have low levels of copy number alterations and low epithelial-mesenchymal transition scores, which are assumed to be related to P16 overexpression, negative ER/PR, and wild-type P53 (Stolnicu 2019). NHPVA has an aggressive phenotype and distinct molecular features (Karamurzin 2015). There is a strong correlation between HPV-associated pathogenesis and morphology in HPVA. *P16* immunohistochemistry is an effective indirect test for HR-HPV infection (Stolnicu 2018). Approximately 95% of HPVA samples exhibit diffuse block-type or every-cell staining (overexpression) (Stolnicu 2018). Importantly, the *P16* results may not be reproducible using old or poorly preserved tissue blocks (Stolnicu 2018). PCR may be used to confirm HPV infection, but its sensitivity and specificity are questionable because the analysis may underperform in archived formalin-fixed tissues. Moreover, PCR does not specifically confirm the presence of HPV within neoplastic cells (Mills et al. 2017). Therefore, it is necessary to identify more credible biomarkers for the complementary ECA diagnosis and prognosis and to provide superior therapeutic strategies for ECA cases.

Transforming acidic coiled-coil protein 3 (TACC3), a member of the TACC family is encoded by the TACC3 gene in 4*P16*.3 (He 2016). As a spindle regulatory protein, TACC3 has a conserved TACC domain at its C-terminus, which plays an important role in its alignment with tubulins and in promoting effective elongation of microtubules during mitosis (Gergely 2000; Ha et al. 2013a; 2017; Mahdipour 2015; Piekorz 2002). An increasing number of studies indicate that, abnormal expression of TACC3 may play an oncogenic role, leading to multiple spindle formation, cellcycle arrest, cell death, and epithelial-mesenchymal transformation (EMT) (Ha et al. 2013b; Huang 2015; Peters 2005; Song 2018; Yun 2015). Numerous studies have indicated that TACC3 is overexpressed in multiple solid tumors (Bhosale 2016) including ovarian cancer (Lauffart 2005), glioblastoma (Duncan 2010), esophageal squamous cell carcinoma (Huang 2015), hepatocellular carcinoma (Nahm 2016; Zhou 2015), gastric carcinoma (Yun 2015), and non-small cell lung cancer (Jung 2006). The fifibroblast growth factor receptor gene 3 and transforming acidic coiled-coil protein-3 (*FGFR3-TAAC3*) fusion gene promotes cancer cell development in some cancer types by promoting cell proliferation (Parker 2013; Capelletti 2014; Yuan 2014; Du 2016). However, the clinical significance of TACC3 in different histologic types of ECA has not yet been reported.

To identify a novel complementary diagnostic and prognostic biomarker for ECA, we have characterized the expression and clinical significance of TACC3 in ECA for the first time in a large cohort of clinical ECA samples. The association between TACC3 expression and clinical pathological clinicopathological parameters was further analyzed. We also assessed the diagnostic performance of TACC3 in ECA relative to other detection methods. Our datas suggest TACC3 as a novel complementary diagnostica,prognostic biomarker and a potential therapeutic target for patients with ECA.

## Materials and methods

### Patients and samples

This study protocol was approved by the Institutional Ethical Board of Sun Yat-sen University Cancer Center. Documented pathological specimens embedded in paraffin, obtained from January 2010 and December 2014, were acquired from 264 ECA cases for analysis, including 239 HPVA and 25 HPVA cases. At the same time, the pathological and clinical data for these patients were collected from patient records. The enrolled patients were aged 19–76 years (average, 65.4) and the median follow-up period was 65.4 months.

### Tissue microarray (TMA) construction and immunohistochemistry (IHC)

Tumorous ECA and adjacent non-tumorous tissues were sampled for TMA. TMA blocks were sectioned at the 4-μm, followed by IHC staining. Then, each slide was deparaffinized with xylene and ethanol, followed by treatment with 3% hydrogen peroxide in methanol. Slides were blocked with avidin–biotin at 4 °C overnight, followed by incubation with antibodies against TACC3 (ab134154, Abcam), P16 (Roche, Germany), MSH2 (ZA0622, Zhongshan, China), MSH6 (Roche (SP93), Germany), MLH1 (Roche (M1), Germany), PMS2 (Dako (EP51), Germany), and Ki-67 (ZA0502, Zhongshan, China). Subsequently, slides were washed thrice with PBS, and further incubated with biotinylated goat anti-mouse antibodies, followed by DAKO liquid 3,3′-diaminobenzidine tetrahydro-chloride (DAB) staining and Mayer’s hematoxylin counterstaining. The staining was independently evaluated by two experienced pathologists. The presence of block-like, diffuse staining in each core indicated positive P16 staining, while patchy or no staining was scored as negative staining. MSH2/MSH6/MLH1/PMS2 were interpreted as positive if ≥ 1% of the tumor cell nuclei were positive. For samples with positive TACC3 and P16 staining, the scores were rated defined as: 0, < 5% cells with positive staining; 1, 5–24% cells with positive staining; 2, 25–49% cells with positive staining; 3, 50–74% cells with positive staining; and 4, 75–100% cells with positive staining. The intensity of positive staining was scored as: 0, negative; 1, weak; 2, moderate; and 3, strong. The final score was determined by multiplying the percentage score by the intensity score. The best cut-off values for all variables were determined using X-tile (Camp et al. 2004): age (37 years), tumor size (4.5 cm), TACC3 (3.7), and Ki-67 (12.5%).

### HPV subtypes

PCR was performed for assaying HPV subtype in tumors that were not represented in tissue microarrays as previously described (Hodgson, et al. 2019). The Roche Cobas 4800 system (Pleasanton, CA,USA) was used for HPV detection, which evaluates for the presence of 14 types of HPV DNA: 16, 18, 31, 33, 35, 39, 45, 51, 52, 56, 58, 59, 66, and 68.

### Statistical analysis

SPSS (version 25.0; SPSS, Chicago, IL, USA) was used for analyses. Significant differences in the expression of TACC3 were determined by Student’ t-test. Data displayed in the bar and column charts are expressed as the mean ± standard error of the mean (SEM). Associations between TACC3 expression and clinicopathological characteristics were analyzed using Fisher’s exact test or Pearson’s chi-square test. The scatter plot shows the correlations between TACC3, P16, and Ki-67. In addition, receiver operating characteristic (ROC) curves were plotted, and the area under the curve (AUC) values together with the corresponding 95% confidence interval (95% CI), were obtained to evaluate sensitivity and specificity. Survival was determined using Kaplan–Meier analysis and survival-related factors were identified using the Cox proportional-hazards regression model. In multivariate analysis, the covariates age, FIGO stage, tumor size, histologic type, stromal invasion, nerve invasion,lymphovascular invasion (LVI), lymph-node metastasis (LNM), parametrium invasion, surgical margin, and P16 expression were compared with the endpoint OS; the covariates FIGO stage, tumor size, histologic type, stromal invasion, nerve invasion, LVI, LNM, parametrium invasion, surgical margin, and P16 expression were compared with the endpoint DFS. The P values of the above variables in univariate analysis were < 0.05. Differences were considered significant when P values were less than 0.05.

## Results

### TACC3 is overexpressed in ECA

**Fig. 1:**
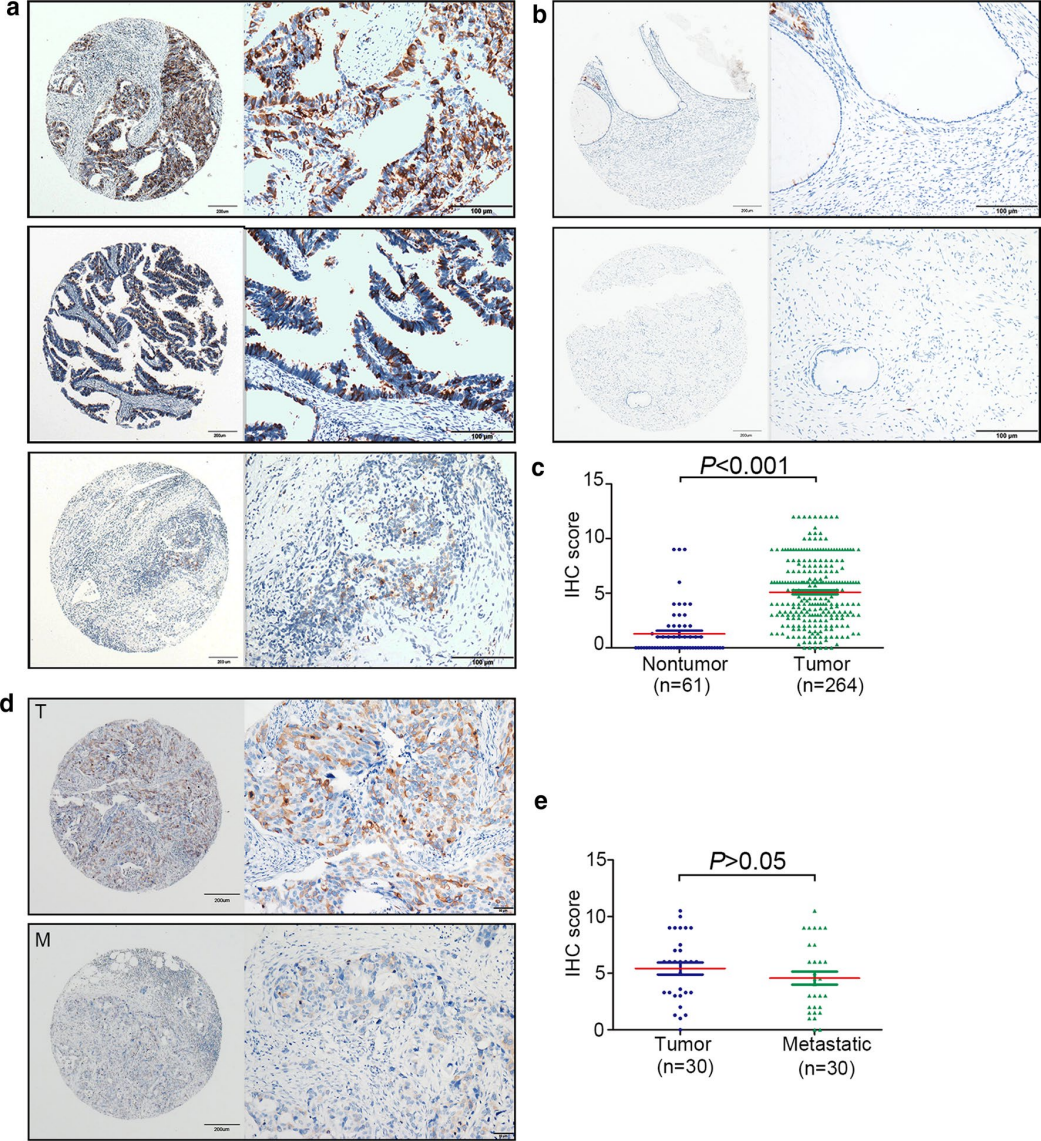
Overexpression of TACC3 in endocervical adenocarcinoma (ECA) detected by immunohistochemistry (IHC). **a** Representative staining for TACC3 in a tissue microarray (TMA) cohort. Representative images of strong, moderate,and weak intensity staining for tumor tissues are shown. **b** Representative IHC images of positive and negative non-tumor tissues are presented. **c** According to IHC scores of the TMA cohort, TACC3 expression in ECA was significantly higher than that in non-tumorous tissues. **d** TACC3 expression in 30 ECA cases with lymph-node metastasis (LNM). Representative graphs are shown for primary tumor (T) and metastatic (M) lesions. **e** Comparison of TACC3 levels between primary tumor and metastatic lesions. Quantitative data are presented as the mean ± standard deviation (SD)

To confirm the expression profile of TACC3 in ECA, 264 archived paraffin-embedded ECA samples were collected and constructed into a TMA cohort along with clinical and pathological information. By IHC, 61.4% of ECA samples and 13.1% of normal samples stained for TACC3. These datas suggest that TACC3 overexpression may contribute to tumor progression. Representative IHC images of TACC3 expression in the tumor are shown with weak, moderate, and strong staining in Fig. 1a. TACC3 was mainly located in the cytoplasm, and its level in ECA was significantly increased relative to non-tumorous samples (Fig. 1b and c). To further evaluate the expression of TACC3 in another TMA cohort, samples from 30 ECA patients with lymph-node metastasis (LNM) were collected. Representative IHC images of TACC3 expression in LNM lesions and the corresponding primary lesions are shown in Fig. 1d. We observed no significant difference between the lymph-node metastases and the primary lesions (*P* > 0.05) (Fig. 1e). Consistently, *TACC3* mRNA expression was was not markedly increased in N0 cervical squamous cell carcinoma (CESC) tissues compared with N1 CESC tissues from The Cancer Genome Atlas (TCGA) dataset (Additional file 1: Figure S1B). Collectively, our data indicate that TACC3 is overexpressed in ECA compared to non-tumorous tissues.

### TACC3, P16, and Ki-67 expression in HPVA and NHPVA

**Fig. 2:**
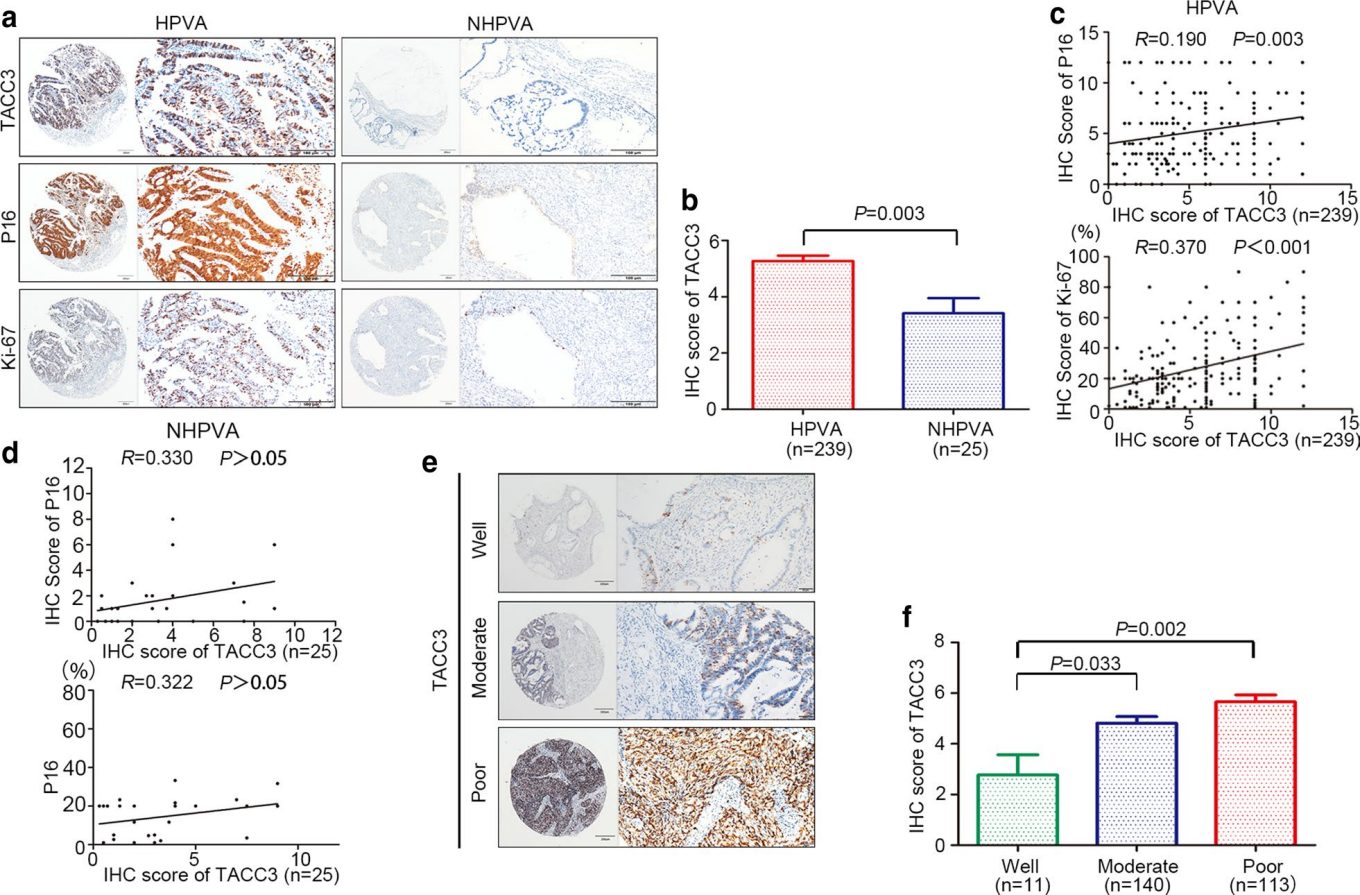
Relationship between TACC3, , and Ki-67 expression in human papillomavirus-associated (HPVA) and non-HPVA (NHPVA) cases. **a** Representative immunohistochemistry (IHC) staining for TACC3, P16, and Ki-67 expression in HPVA and NHPVA. **b** According to the IHC scores of HPVA and NHPVA, TACC3 expression in HPVA was significantly higher than that in NHPVA. **c** Relationship between TACC3 expression and that of P16 and Ki-67 in HPVA. **d** Relationship between TACC3 expression and that of P16 and Ki-67 in NHPVA. **e** Representative images of TACC3 expression in well differentiated, moderately differentiated and poorly differentiated tumors. **f** Comparison of TACC3 levels in ell differentiated, moderately differentiated and poorly differentiated tumors. Quantitative data are presented as the mean ± standard deviation (SD)

Next, we analyzed the expression profile of *TACC3* in differenthistological subtypes of ECA. Additional file 5: Table S1 presents the clinicopathological data for the HPVA and NHPVA cases. By IHC, 64.0% of ECA samples and 36.0% of normal samples were positive for TACC3 in HPVA and NHPVA. These datas suggest that overexpressed TACC3 may perform diverse biological functions in different histological subtypes. Representative IHC images for TACC3, P16, and Ki-67 in the HPVA and NHPVA subtypes are shown in Fig. 2a. TACC3 expression was remarkably higher in HPVA than in NHPVA (Fig. 2b). Positive correlations were found between high TACC3 expression and P16 positive expression as well as between high TACC3 expression and high Ki-67 expression in HPVA (r = 0.190, *P* = 0.003; r = 0.370, *P* < 0.001, respectively), but no significant associations were found in *NHPA* (Fig. 2c and d). Representative IHC images of TACC3 expression in well-differentiated,moderately differentiated and poorly differentiated tumors are shown in Fig. 2e. TACC3 expression in poorly differentiated tumors tumor was remarkably higher than that in well differentiated tumors (Fig. 2f),which was consistent with the TCGA data (Additional file 1: Figure S1B).

### Effect of TACC3 on overall survival (OS) and disease-free survival (DFS) of patients with ECA

**Fig. 3:**
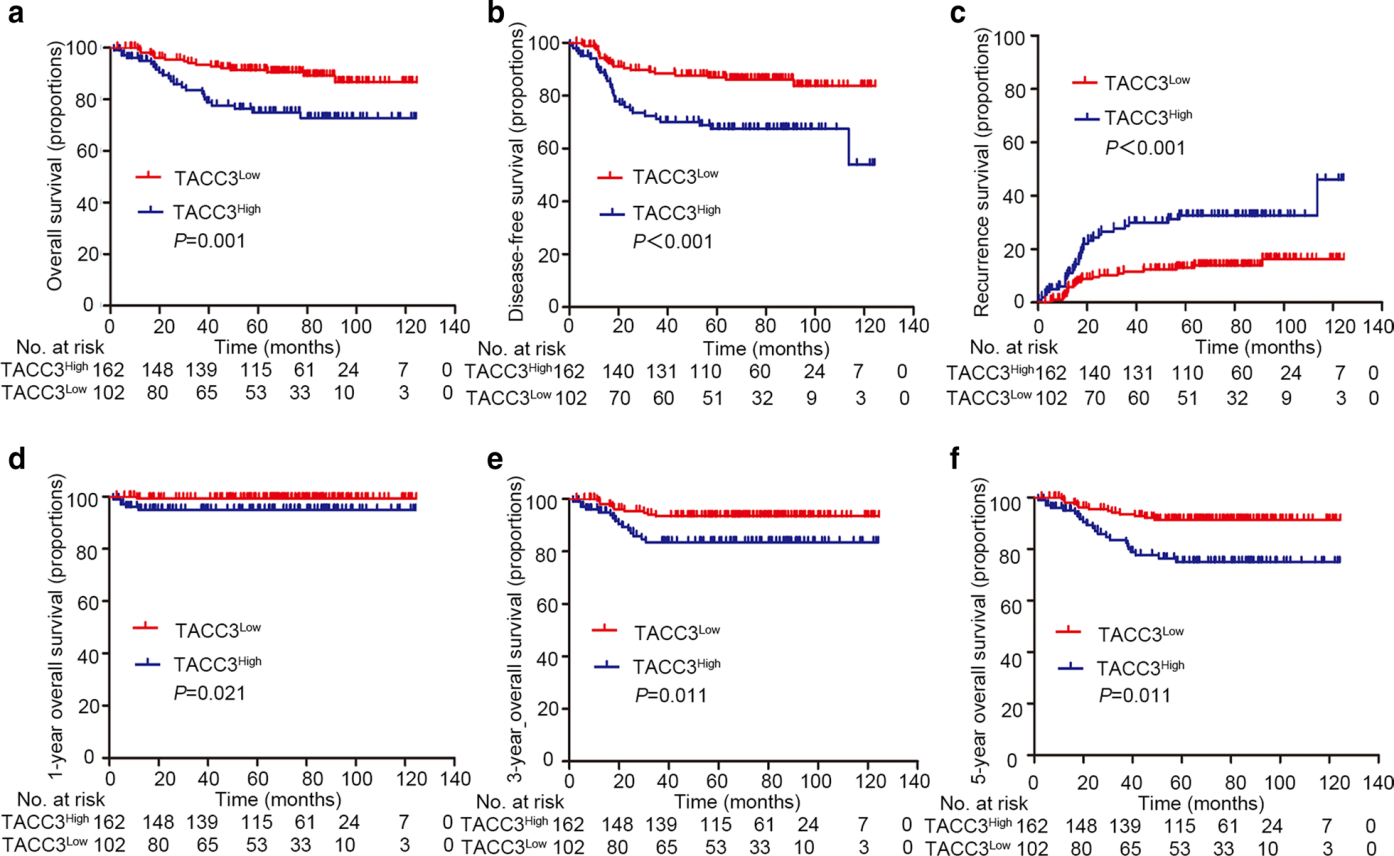
Association of TACC3 expression and survival of patients with endocervical adenocarcinoma (ECA). **a** Correlation of TACC3 expression and overall survival determined in a tissue microarray (TMA) cohort including 264 patients by Kaplan–Meier analysis. **b** Disease-free survival of the same TACC3 TMA cohort. **c** Curve for relapse evaluated according to TACC3 expression level. **d**–**f** 1-, 3- and 5-year overall survival (OS)of the same TACC3 TMA cohort. The life table is shown below each graph

**Fig. 4:**
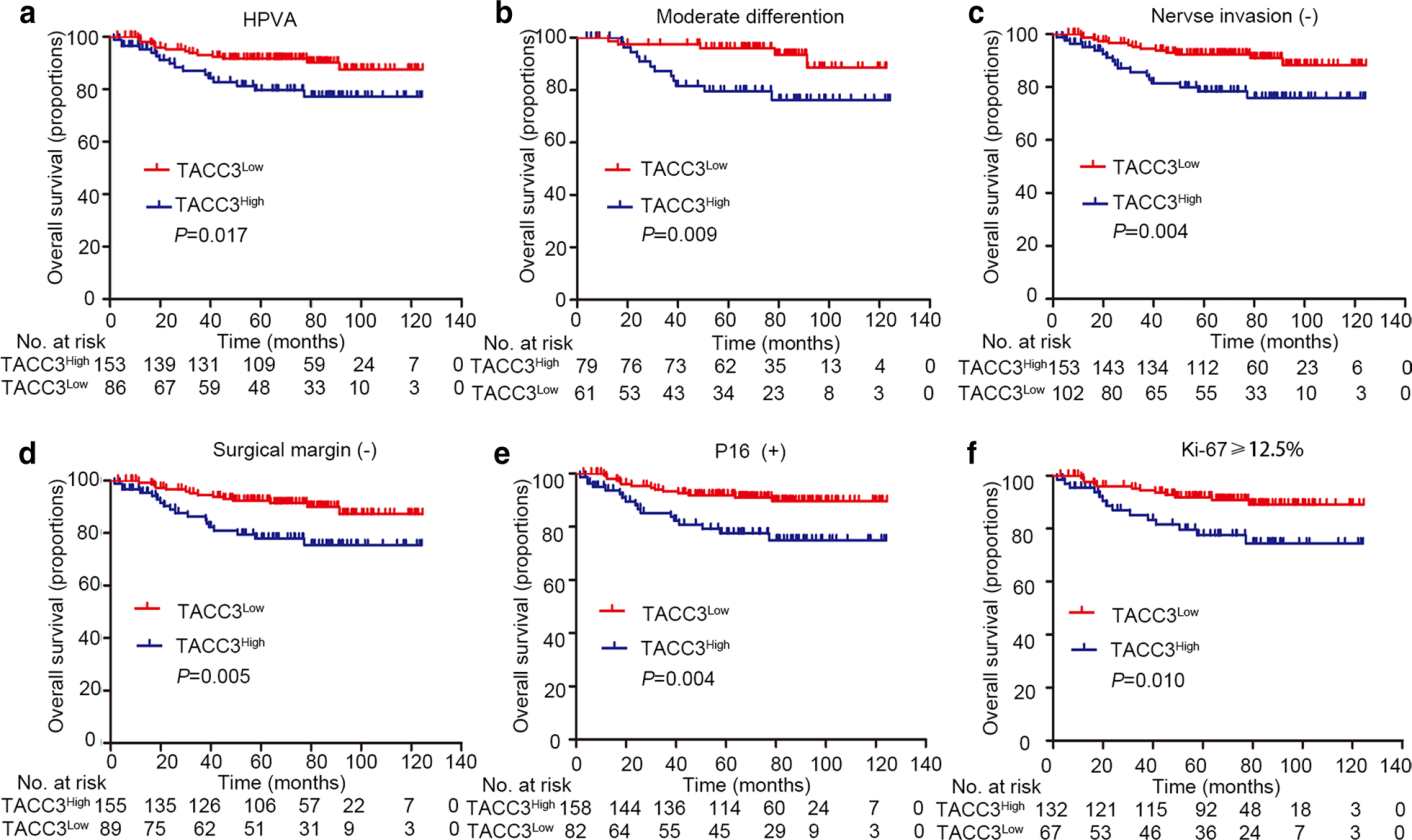
Stratified analysis of TACC3 expression related to overall survival. Correlations between TACC3 expression and overall survival in the indicated groups

To better understand the clinical implications of TACC3 expression in ECA, the associations of TACC3 expression levels with clinicopathological factors for ECA cases were examined. Based on the TACC3 IHC staining score threshold of 3.7, patients with ECA were divided into two groups: high *vs. * low TACC3 expression (Additional file 2: Figure S2A). High TACC3 expression was detected in 61.4% (162/264) of the patients. Increased TACC3 levels were significantly associated with tumor differentiation (*P* = 0.004), histological type (*P* = 0.006), parametrium invasion (*P* = 0.040), nerve invasion (*P* = 0.003), surgical margin (*P* = 0.012), P16 (*P* < 0.001), and Ki-67 (*P* = 0.004) (Table 1). Similarly, TCGA data showed that TACC3 expression was associated with proliferation-related parameters including E2F targets, G2M checkpoint, G2 pathway, and proliferation-associated biomarkers (Additional file 2: Figures S1D-F and S2B). Subsequently, the value of TACC3 in predicting the prognosis of ECA was analyzed. As suggested by Kaplan–Meier analysis, cases with high TACC3 expression were associated with reduced OS (*P* = 0.001) reduced and at 1-year (*P* = 0.021), 3-year (*P* = 0.011), and 5-year OS (*P* = 0.011) relative to those with low TACC3 expression. In addition, high TACC3 expression was correlated with dismal DFS (*P* < 0.001) as well as recurrence (*P* < 0.001) of ECA (Fig. 3). Furthermore, stratified survival analysis verified the prognostic significance of TACC3. TACC3 expression was correlated with multiple OS-related pathological factors (Fig. 4).

**Table 1:** Correlation of clinicopathological parameters and TACC3 expression (n = 264)

Variable	TACC3 expression			
	All cases	Low	High	*P* value^a^
Age (years)				0.114
< 37	31	16 (51.6%)	15 (48.4%)	
≥ 37	233	86 (36.9%)	147 (63.1%)	
Figo stage				0.085
I	186	66 (35.5%)	120 (64.5%)	
II	67	30 (44.8%)	37 (55.2%)	
III	8	3 (37.5%)	5 (62.5%)	
IV	3	3 (100.0%)	0 (0.0%)	
Tumor size (cm)				0.414
< 4.5	221	83 (37.6%)	138 (62.4%)	
≥ 4.5	43	19 (44.2%)	24 (55.8%)	
Histological type				**0.006**
HPVA	239	86 (36.0%)	153 (64.0%)	
NHPVA	25	16 (64.0%)	9 (36.0%)	
Differentiation				**0.004**
Well	11	8 (72.7%)	3 (27.3%)	
Moderate	140	61 (43.6%)	79 (56.4%)	
Poor	113	33 (29.2%)	80 (70.8%)	
Stromal invasion				0.120
< 1/3	67	27 (40.3%)	40 (59.7%)	
1/3–2/3	81	24 (29.6%)	57 (70.4%)	
≥ 2/3	116	50 (43.1%)	66 (56.9%)	
Nerve invasion				**0.003**
Negative	238	85 (35.7%)	153 (64.3%)	
Positive	26	17 (65.4%)	9 (34.6%)	
LVI				0.800
None (0)	180	73 (40.6%)	107 (59.4%)	
Focal (1–4)	53	19 (35.8%)	34 (64.2%)	
Moderate (5–9)	18	6 (33.3%)	12 (66.7%)	
Extensive (≥ 10)	13	4 (30.8%)	9 (69.2%)	
LNM				0.395
Negative	204	76 (37.3%)	128 (62.7%)	
Positive	60	26 (43.3%)	34 (56.7%)	
Parametrium invasion				**0.040**
Negative	242	89 (36.8%)	153 (63.2%)	
Positive	22	13 (59.1%)	9 (40.9%)	
Surgical margin				**0.012**
Negative	244	89 (36.5%)	155 (63.5%)	
Positive	20	13 (65.0%)	7 (35.0%)	
HPV subtype				0.108
HPV 16	67	19 (28.4%)	48 (71.6%)	
HPV 18	72	25 (34.7%)	47 (65.3%)	
Other subtypes	18	8 (44.4%)	10 (55.6%)	
Not available	1	0 (0.0%)	1 (100.0%)	
Negative	106	50 (47.2%)	56 (52.8%)	
MMR				0.278
dMMR	20	10 (50.0%)	10 (50.0%)	
pMMR	244	92 (37.7%)	152 (62.3%)	
P16				**0.000**
Negative	24	20 (83.3%)	4 (16.7%)	
Positive	240	82 (34.2%)	158 (65.8%)	
Ki67				**0.004**
< 12.5	65	35 (53.8%)	30 (46.2%)	
≥ 12.5	199	67 (33.7%)	132 (66.3%)	

Bold values indicate* P* value was less than 0.05

^a^Chi-square test

HPVA, HPV-associated adenocarcinoma; NHPVA, nonHPV-associated adenocarcinoma; LVI, lymph vascular invasion; LNM, lymph node metastasis; MMR, mismatch repair; dMMR, deficient mismatch repair; pMMR, proficient mismatch repair; other subtypes, HPV45, HPV16/18, HPV18/45, HPV73/35/81, HPV53/56/66, HPV26/51/82, HPV18/39/59/68

Cox regression analysis indicated that TACC3 expression is a prognostic factor, along with age, FIGO stage, tumor size, histologic type, stromal invasion, nerve invasion, LVI, LNM, parametrium invasion, surgical margin, and P16 expression (Table 2). Multivariate survival analysis suggested that TACC3 is an independent factor for decreased OS (HR = 2.280, 95% CI: 1.087–4.783, *P* = 0.029) and DFS (HR = 2.265, 95% CI: 1.232–4.166, *P* = 0.009) (Tables 2 and 3).

**Table 2:** Univariate and multivariate analyses of clinicopathological parameters and TACC3 expression for overall survival (n = 264)

Variables	Univariate analysis		Multivariate analysis	
	HR (95% CI)	*P*	HR (95% CI)	*P*
Age (< 37 vs. ≥ 37 years)	0.381 (0.180–0.802)	**0.011**	0.678 (0.280–1.639)	0.388
Figo stage (I vs. II vs. III vs. IV)	2.383 (1.585–3.581)	**0.000**	1.116 (0.699–1.782)	0.645
Tumor size (cm) (< 4.5vs. ≥ 4.5)	2.414 (1.222–4.768)	**0.011**	1.079 (0.487–2.390)	0.851
Histological type (HPVA vs. NHPVA)	0.293 (0.138–0.621)	**0.001**	0.610 (0.228–1.632)	0.325
Differentiation (Well vs. Moderate vs. Poor)	1.427 (0.803–2.535)	0.226		
Stromal invasion (< 1/3 vs.1/3–2/3vs. ≥ 2/3)	3.581 (2.004–6.400)	**0.000**	1.832 (0.953–3.522)	0.070
Nerve invasion (Negative vs. Positive)	3.990 (1.884–8.450)	**0.000**	0.653 (0.246–1.730)	0.391
LVI (None vs. Focal vs. Moderate vs. Extensive)	2.061 (1.550–2.741)	**0.000**	1.740 (1.200–2.524)	**0.003**
LNM (Negative vs. Positive)	7.270 (3.832–13.794)	**0.000**	3.100 (1.381–6.959)	**0.006**
Parametrium invasion (Negative vs. Positive)	5.650 (2.581–12.367)	**0.000**	1.345 (0.514–3.516)	0.546
Surgical margin (Negative vs. Positive)	3.401 (1.499–7.716)	**0.003**	1.123 (0.430–2.934)	0.813
HPV subtype (HPV 16 vs. HPV18 vs. Other types vs. Not available vs. Negative)	0.845 (0.607–1.177)	0.321		
MMR (dMMR vs. pMMR)	0.918 (0.283–2.981)	0.886		
P16 (Negative vs. Positive)	0.332 (0.152–0.723)	**0.006**	0.584 (0.214–1.598)	0.295
Ki67 (< 12.5vs. ≥ 12.5)	0.614 (0.316–1.196)	0.152		
TACC3 (Low vs. High)	2.727 (1.440–5.165)	**0.002**	2.280 (1.087–4.783)	**0.029**

Bold values indicate* P* value was less than 0.05

HR, hazard ratio; CI, confidence interval. HPVA, HPV-associated adenocarcinoma; NHPVA, nonHPV-associated adenocarcinoma; LVI, lymph vascular invasion; LNM, lymph node metastasis; MMR, mismatch repair; dMMR, deficient mismatch repair; pMMR, proficient mismatch repair; other subtypes, HPV45, HPV16/18, HPV18/45, HPV73/35/81, HPV53/56/66, HPV26/51/82, HPV18/39/59/68

**Table 3:** Univariate and multivariate analyses of clinicopathological parameters and TACC3 expression for Disease-free survival (n = 264)

Variables	Univariate analysis		Multivariate analysis	
	HR (95% CI)	*P*	HR (95% CI)	*P*
Age (< 37 vs. ≥ 37 years)	0.530 (0.266–1.056)	0.071		
Figo stage (I vs. II vs. III vs. IV)	1.941 (1.330–2.832)	**0.001**	1.055 (0.690–1.614)	0.804
Tumor size (cm) (< 4.5 vs. ≥ 4.5)	2.303 (1.280–4.145)	**0.005**	1.138 (0.581–2.228)	0.707
Histological type (HPVA vs. NHPVA)	0.310 (0.162–0.592)	**0.000**	0.536 (0.241–1.192)	0.126
Differentiation (Well vs. Moderate vs. Poor)	1.266 (0.778–2.062)	0.343		
Stromal invasion (< 1/3 vs.1/3–2/3 vs. ≥ 2/3)	2.363 (1.557–3.585)	**0.000**	1.468 (0.908–2.373)	0.117
Nerve invasion (Negative vs. Positive)	2.846 (1.426–5.680)	**0.003**	0.659 (0.275–1.581)	0.350
LVI (None vs. Focal vs. Moderate vs. Extensive)	1.600 (1.214–2.108)	**0.001**	1.367 (0.975–1.917)	0.070
LNM (Negative vs. Positive)	4.631 (2.685–7.987)	**0.000**	2.665 (1.350–5.262)	**0.005**
Parametrium invasion (Negative vs. Positive)	4.099 (1.987–8.456)	**0.000**	1.495 (0.635–3.523)	0.357
Surgical margin (Negative vs. Positive)	2.687 (1.264–5.712)	**0.010**	1.019 (0.435–2.390)	0.965
HPV subtype (HPV 16 vs. HPV18 vs. Other types vs. Not available vs. Negative)	0.846 (0.637–1.123)	0.247		
MMR (dMMR vs. pMMR)	0.969 (0.350–2.686)	0.952		
P16 (Negative vs. Positive)	0.335 (0.172–0.652)	**0.001**	0.669 (0.293–1.530)	0.341
Ki67 (< 12.5vs. ≥ 12.5)	0.784 (0.431–1.426)	0.426		
TACC3 (Low vs. High)	2.621 (1.517–4.530)	**0.001**	2.265 (1.232–4.166)	**0.009**

Bold values indicate* P* value was less than 0.05

HR, hazard ratio; CI, confidence interval. HPVA, HPV-associated adenocarcinoma; NHPVA, nonHPV-associated adenocarcinoma; LVI, lymph vascular invasion; LNM, lymph node metastasis; MMR, mismatch repair; dMMR, deficient mismatch repair; pMMR, proficient mismatch repair; other subtypes, HPV45, HPV16/18, HPV18/45, HPV73/35/81, HPV53/56/66, HPV26/51/82, HPV18/39/59/68

### Diagnostic performance of TACC3 in ECA relative to other detection methods.

**Fig. 5:**
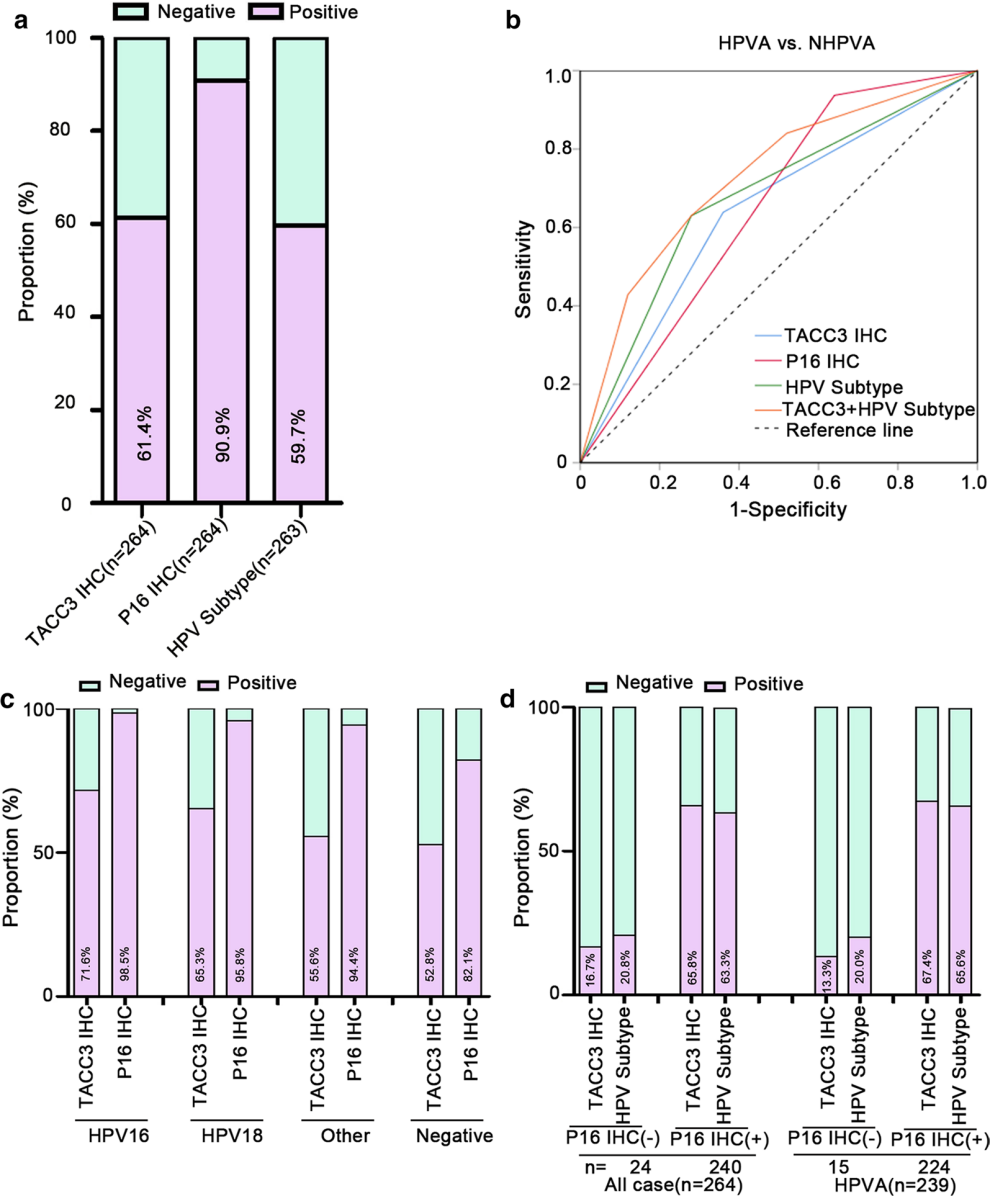
Diagnostic performance of tests for patients with endocervical adenocarcinoma (ECA). **a** Positive rates of TACC3, P16, and HPV subtypes in ECA cases. **b** ROC curve analyses of TACC3, P16, HPV subtypes, and the panel of TACC3 + HPV subtypes. **c** Positive rates of TACC3 and P16 in ECA cases with different HPV subtypes. **d** Positive rates of TACC3 and HPV subtypes in patients with ECA in P16-negative and P16-positive subgroups. TACC3 immunohistochemistry (IHC), TACC3 protein detected by IHC; P16 IHC, P16 protein detected by IHC; HPV subtypes, HPV subtypes detected by PCR

To distinguish HPVA from NHPVA*,* the expression of TACC3 and P16 proteins was detected by IHC, and HPV subtypes were identified by PCR. The positive rates of TACC3, P16, and HPV subtypes were 61.4%, 90.9%, and 59.7%, respectively (Fig. 5a). ROC curve analyses suggested that TACC3, P16 and HPV subtypes were similarly able to distinguish HPVA from NHPA (AUC = 0.640, sensitivity = 64.0%, specificity = 64.0%; AUC = 0.649, sensitivity = 93.7%, specificity = 36.0%; AUC = 0.675, sensitivity = 63.0%, specificity = 72.0%, respectively) (Additional file 5: Table S2). ROC curve analyses showed that the combination of TACC3 expression with HPV subtypes improved diagnostic performance over those of TACC3, P16, and HPV subtypes alone (Fig. 5b and Additional file 1: Table S2). The correlation of TACC3, P16, and HPV subtypes was also assessed (Fig. 5c and d). We found that 59.7% (157/263) of ECA cases were associated with HPV, particularly with HPV strains 16 and 18. For different HPV subtypes, the positivity of P16 for the diagnosis of ECA was much higher than that of TACC3 (Fig. 5c). We observed that TACC3 positivity in all cases, and IHC revealed P16 positivity in HPVA (all case, 65.8%; HPVA, 67.4%), similar to the positivity of HPV subtypes (all case, 63.3%; HPVA, 65.6%). The positivity of TACC3 and HPV subtypes was much higher in the P16-positive group than in the P16-negative group (Fig. 5d). Therefore, our data indicate that TACC3 has promise as a complementary diagnostic marker for patients with ECA.

## Discussion

Currently, the relative prevalence of ECA has increased to 10–25% of all cervical carcinomas in developed countries, predominantly due to the impact of cytology-based screening on the detection and treatment of squamous precancers (Smith et al. 2000; Adegoke et al. 2012). According to the IECC, ECA can be classified as HPVA or NHPVA, based on morphological features. HPVA is associated with significantly better disease-free and disease-specific survival than NHPVA (Stolnicu 2019). To improve ECA prognostic outcomes and better stratify HPVA and NHPVA, it is necessary to identify more credible biomarkers for disease diagnosis and prognosis. We have shown that TACC3, a spindle regulatory protein is overexpressedin ECA, with overexpression associated with poor OS and DFS. Moreover, multivariate analysis revealed TACC3 as an independent prognostic predictor. Additionally, TACC3 was used as a complementary diagnostic marker for ECA. To our knowledge, the present work is the first to reveal the clinical implication pf TACC3 in ECA.

Abnormal expression of TACC3 is detected in human malignancies, and its role has been extensively investigated (Wang 2017a). *TACC3* is overexpressed in human malignancies and has oncogenicproperties (Wang 2017a). In addition, TACC3 overexpression is associated with a dismal prognostic outcome (Song 2018; Nahm 2016; Li 2017). TACC3 was previously suggested to piay a vital role in epidermal growth factor (EGF) mediated EMT, which represents a promising therapeutic strategy for the treatment of cervical carcinoma and is involved in the EGF/EGF receptor (EGFR) signal transduction pathway (Ha et al. 2013c). Nonetheless, the expression pattern and clinical value of ECA remain unknown. Our study showed that TACC3 levels were upregulated in ECA, especially in HPVA. ECA cases with high TACC3 expression were associated with shortened OS and DFS compared to those with low TACC3 expression. The above results indicate that TACC3 may serve as a novel biomarker for predicting the prognosis of ECA cases. In addition, TACC3 protein levels were upregulated in the moderately and poorly differentiated samples compared with the well-differentiated samples. In our study, higher TACC3 expression was correlated with Ki-67 expression, and TCGA data showed that TACC3 expression was associated with the expression of proliferation-related genes. Therefore, it is of interest to determine whether TACC3 is involved in ECA cell differentiation and proliferation.Several studies have attempted to examine the role of TACC3 in tumor development. Ha et al. reported that TACC3 activated the phosphatidylinositol 3-kinase (PI3K)/Akt and extracellular signal-regulated protein kinases (ERKs) signal transduction pathways to accelerate the EMT and proliferation (Ha et al. 2013b). He et al. showed that histone deacetylase inhibitors (HDACIs) decreased TACC3 and inhibited the proliferation and clone-forming capacity of cholangiocarcinoma cells (He 2016). TACC3 knockdown improved tumor cell sensitivity to chemotherapeutics through effective regulation of premature senescence (Yim 2009).

Cell lines that express the *FGFR3-TACC3* fusion are sensitive to FGFR inhibition, and the *FGFR3-TACC3* fusion is a molecular characteristic that is susceptible to FGFR inhibitors (Wang 2017b). This fusion has been described in approximately 2% of CESC cases in Ryo Tamura’s study (Tamura 2018) and case reports (Carneiro 2015). In a previous study that analyzed RNA sequencing data from 4366 primary tumor samples across 13 tumor types, the *FGFR3-TACC3* fusion was detected more frequently in squamous cell carcinoma than adenocarcinoma (Tamura 2018). In line with this previous result, we did not find any *FGFR3-TACC3* fusion-positive cases in a cohort of 37 patients with ECA by RNA fluorescence in situ hybridization (FISH) (Additional file 3: Figure S3). Therefore, *FGFR3-TACC3* fusion may not be associated with ECA tumorigenesis; however, additional samples are needed to confirm this. Targeted therapies against TACC3 are expected to be spotlighted in the future.

TACC3 protein levels were upregulated in HPVA tissues compared with NHPVA tissues.Therefore, it is important to examine the role of TACC3 in HPVA development. In our study, we used PCR and IHC against the P16 protein to assess HPV status. PCR is a complementary test for assessing HPV infection, but its sensitivity and specificity are questionable because it may underperform in archived, formalin-fixed tissues (Mills et al. 2017). Additionally, P16 immunostaining represents an indirect, cost-effective test for viral infection and has been extensively utilized in practical applications. It shows the highest sensitivity, but low specificity because the overexpression of P16 is detected in cases with upregulated cell cycle, such as inflammation or other viral infections. Using ROC curves, P16 immunostaining was the best diagnostic candidate, while TACC3 IHC and HPV subtypes had similar diagnostic associations. However, TACC3 IHC and HPV subtypes can be combined to improve diagnostic effectiveness. When both P16 IHC and HPV subtypes are negative, TACC3 might be positively expressed. In this case, the missed diagnosis of ECA can be avoided to a certain extent, especially in the case of biopsy. Therefore, TACC3 is expected to be a complementary diagnostic marker for the detection of ECA. However,further research is required to confirm this hypothesis.

## Conclusion

In summary, our data demonstrated the upregulation of TACC3 in ECA samples,which predicted unfavorable overall and disease-free survival. The combination of TACC3 and HPV subtypes improved the diagnostic performance of ECA compared with TACC3, P16 or HPV subtypes alone. Collectively, our data identified TACC3 as a novel promising complementary diagnostic and prognostic biomarker for patients with ECA.

## Supplementary Information

**Additional file 1: Figure S1.** Enrichment and expression levels of *TACC3* in The Cancer Genome Atlas (TCGA) database. (A) We used an available online database to evaluate the *TACC3* expression profile, which showed that *TACC3* mRNA expression in cervical squamous cell carcinoma (CESC) was significantly higher than that in non-tumorous tissues. (B-C) *TACC3* mRNA expression was associated with CESC with N stage and different grades. (D-F) High *TACC3* mRNA expression was positively correlated with E2F targets, G2M checkpoint, and G2 pathway.

**Additional file 2: Figure S2.** Bes cut-off values for all variables determined by X-tile in all endocervical adenocarcinoma (ECA) cases. (A) Best cut-off values for TACC3 Determined by X-tile. (B) Protein–protein interaction analysis performed on TACC3. (C) Correlation of histologic types and overall survival determined in a tissue microarray (TMA) cohort including 264 patients by Kaplan–Meier analysis.

**Additional file 3: Figure S3**. No* FGFR3-TACC3* fusion-positive cases were detected in a cohort of 37 patients with endocervical adenocarcinoma (ECA) by RNA fluorescence in situ hybridization (FISH).* FGFR3-TACC3* fusion may not be associated with tumorigenesis (original magnifications 4× and 1000×).

**Additional file 4: Figure S4**. Upstream mechanisms of action of* TACC3 overexpression* in cervical cancer. (A) Genetic alterations were detected in approximately 5% of patients withcervical cancer from The Cancer Genome Atlas (TCGA) cohort (n = 270), and tumors with mutations were extremely rare. (B) Specific CpG islands. (C) The promoter methylation level of* TACC3* in CESC was markedly decreased in CESC tissues compared with normal tissues from the TCGA dataset.

**Additional file 5: Table S1.** Characteristics of patients with the human papillomavirus. **Table S2.** Diagnostic performances of studied testing for ECA patients.

## Data Availability

The data sets supporting the results of this article are included within the article and its additional files.

## Abbreviations

TACC3: Transforming acidic coiled-coil protein3
ECA: Endocervical adenocarcinoma
*HPV*: Human papillomavirus
HPVA: Human papillomavirus associated
NHPVA: Non-HPVA
IECC: International Endocervical Adenocarcinoma Criteria and Classification
HR-HPV: High-risk HPV
EMT: Epithelial–mesenchymal transformation
*FGFR3-TAAC3*: Fifibroblast growth factor receptor gene 3 and transforming acidic coiled-coil protein3
FGFR: Fifibroblast growth factor receptor
TMA: Tissue microarray
IHC: Immunohistochemistry
LVI: Lymphovascular invasion
LNM: Lymph-node metastasis
CESC: Cervical squamous cell carcinoma
EGF: Epidermal growth factor
EGF/EGFR: EGF/EGF receptor
TCGA: The Cancer Genome Atlas
PCR: Polymerase chain reaction
FFPE: Formalin-fixed paraffin-embedded
ROC: Receiver operating characteristic
AUC: Area under the curve
PI3K: Phosphatidylinositol 3-kinase
ERKs: Extracellular signal-regulated protein kinases
