# Supplementary figures and images for: Transforming acidic coiled-coil protein-3: a novel marker for differential diagnosis and prognosis prediction in endocervical adenocarcinoma
Source: Mol Med. 2021 Jun 10;27:60. doi: 10.1186/s10020-021-00298-z (PMC8210387; doi:10.1186/s10020-021-00298-z)

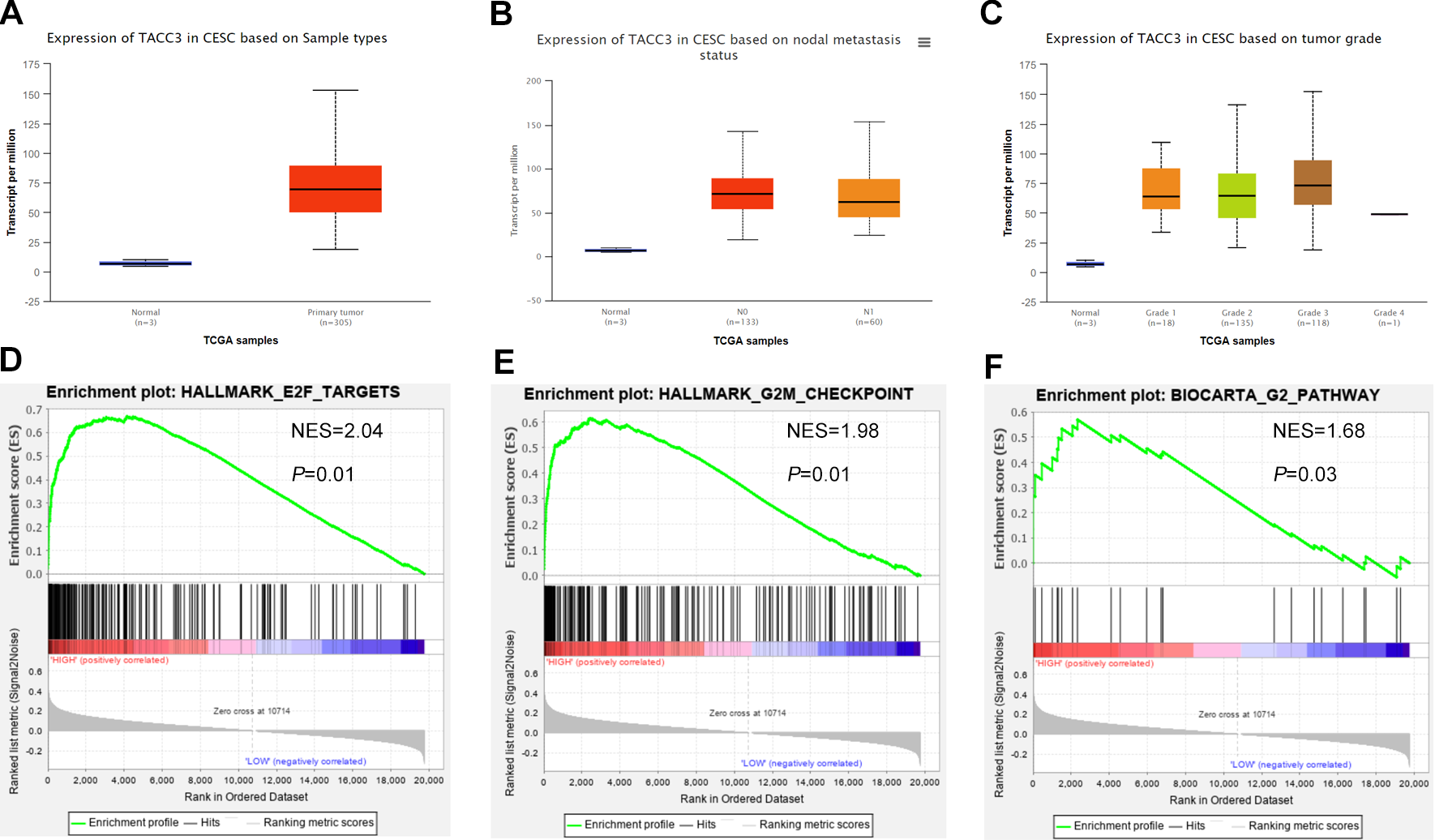

Supplement: Supplementary file 1 — Additional file 1: Figure S1. Enrichment and expression levels of TACC3 in The Cancer Genome Atlas (TCGA) database. (A) We used an available online database to evaluate the TACC3 expression profile, which showed that TACC3 mRNA expression in cervical squamous cell carcinoma (CESC) was significantly higher than that in non-tumorous tissues. (B-C) TACC3 mRNA expression was associated with CESC with N stage and different grades. (D-F) High TACC3 mRNA expression was positively correlated with E2F targets, G2M checkpoint, and G2 pathway. [file 10020_2021_298_MOESM1_ESM.tif]

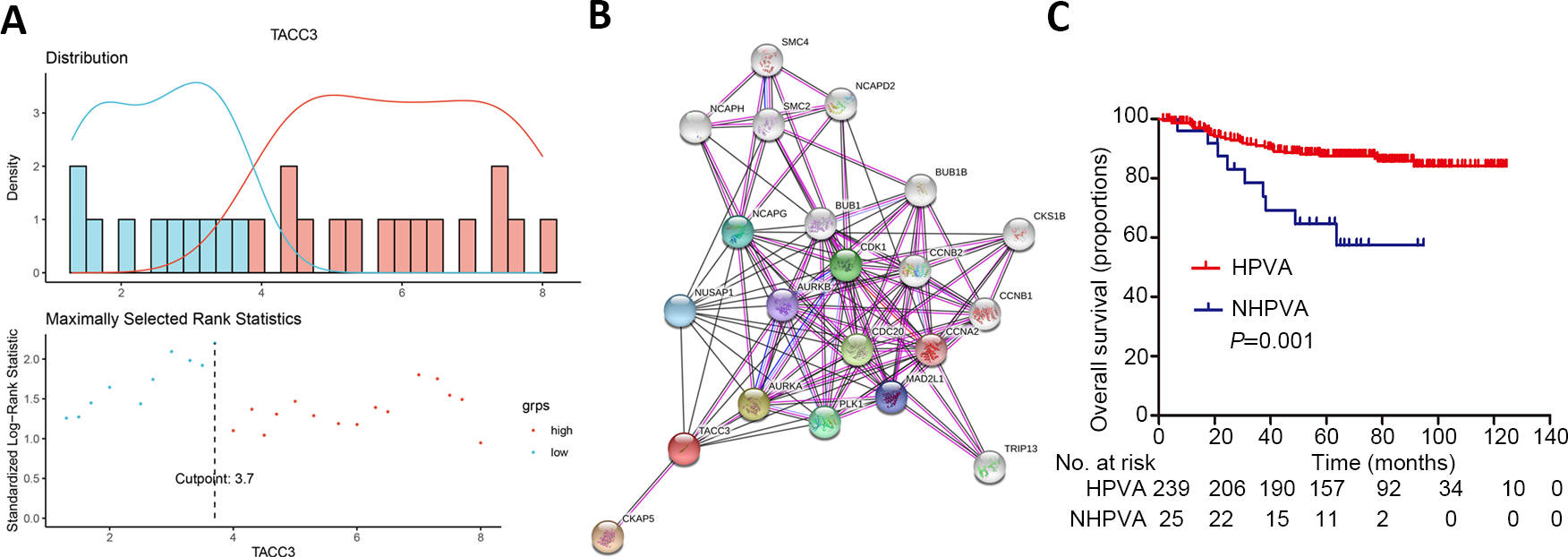

Supplement: Supplementary file 2 — Additional file 2: Figure S2. Bes cut-off values for all variables determined by X-tile in all endocervical adenocarcinoma (ECA) cases. (A) Best cut-off values for TACC3 Determined by X-tile. (B) Protein–protein interaction analysis performed on TACC3. (C) Correlation of histologic types and overall survival determined in a tissue microarray (TMA) cohort including 264 patients by Kaplan–Meier analysis. [file 10020_2021_298_MOESM2_ESM.tif]

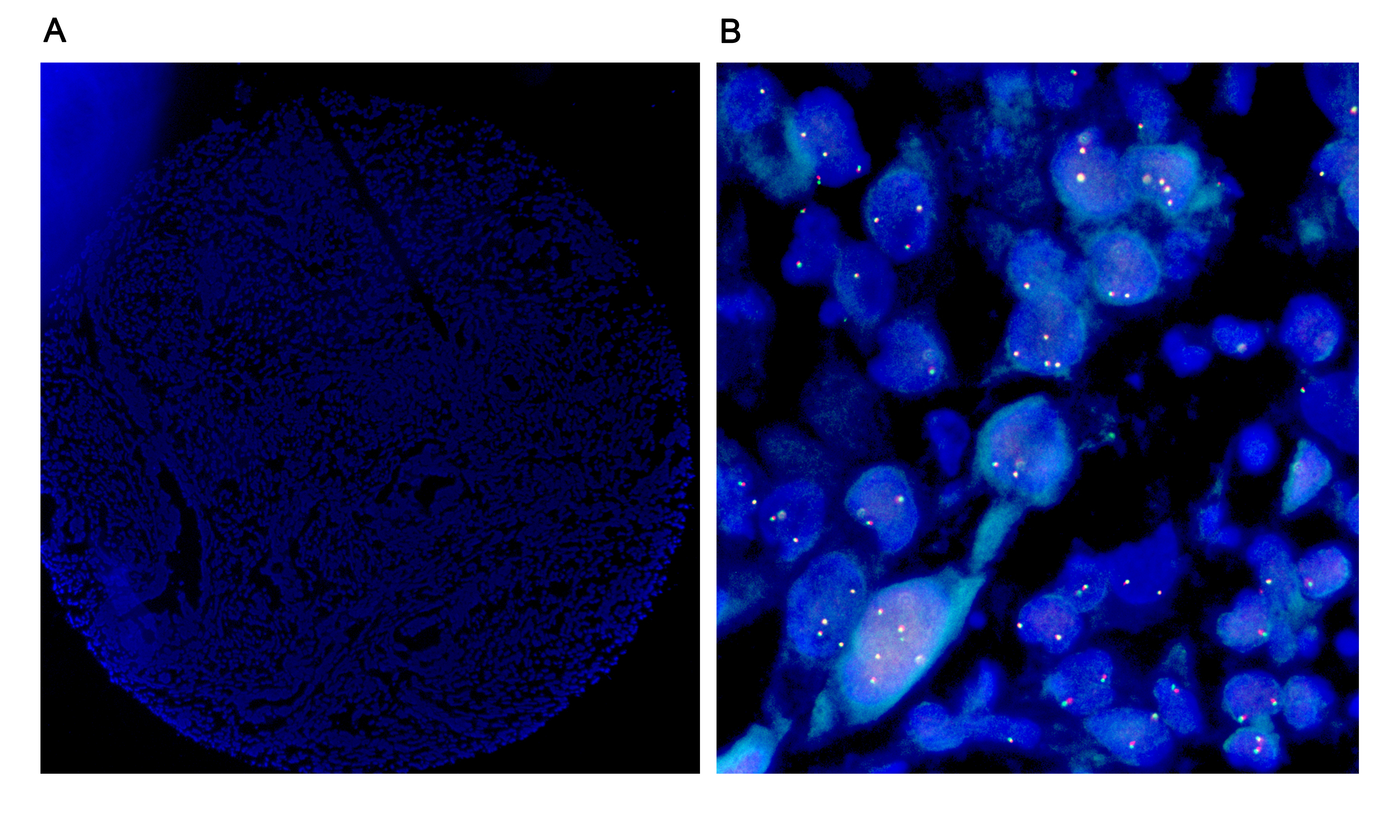

Supplement: Supplementary file 3 — Additional file 3: Figure S3. No FGFR3-TACC3 fusion-positive cases were detected in a cohort of 37 patients with endocervical adenocarcinoma (ECA) by RNA fluorescence in situ hybridization (FISH). FGFR3-TACC3 fusion may not be associated with tumorigenesis (original magnifications 4× and 1000×). [file 10020_2021_298_MOESM3_ESM.jpg]

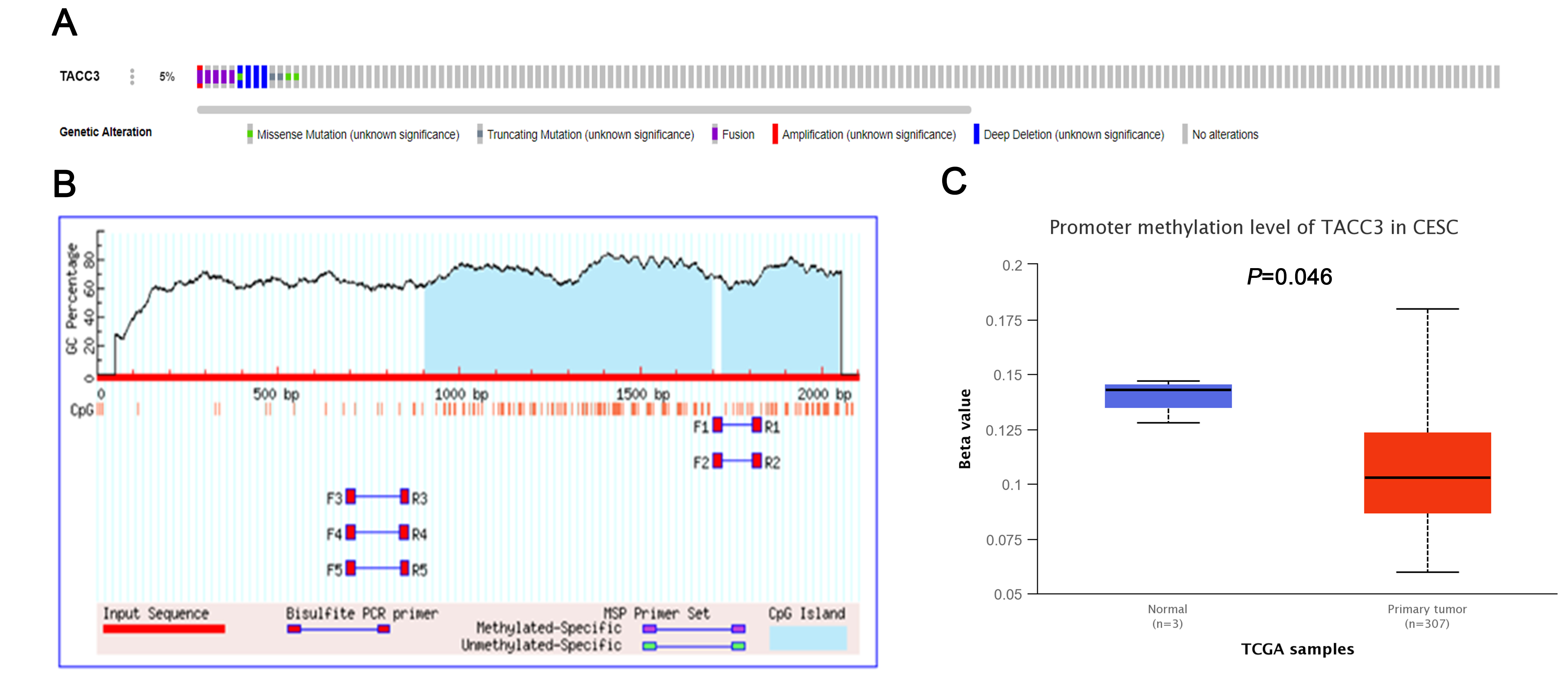

Supplement: Supplementary file 4 — Additional file 4: Figure S4. Upstream mechanisms of action of TACC3 overexpression in cervical cancer. (A) Genetic alterations were detected in approximately 5% of patients withcervical cancer from The Cancer Genome Atlas (TCGA) cohort (n = 270), and tumors with mutations were extremely rare. (B) Specific CpG islands. (C) The promoter methylation level of TACC3 in CESC was markedly decreased in CESC tissues compared with normal tissues from the TCGA dataset. [file 10020_2021_298_MOESM4_ESM.jpg]
