# Supplementary material for: Transforming acidic coiled-coil protein-3: a novel marker for differential diagnosis and prognosis prediction in endocervical adenocarcinoma
Source: Mol Med. 2021 Jun 10;27:60. doi: 10.1186/s10020-021-00298-z (PMC8210387; doi:10.1186/s10020-021-00298-z)
Supplement: Supplementary file 5 — Additional file 5: Table S1. Characteristics of patients with the human papillomavirus. Table S2. Diagnostic performances of studied testing for ECA patients. [file 10020_2021_298_MOESM5_ESM.docx]

**Supplementary Table 1. Characteristics of patients with the human papillomavirus.**

| **Variables** | **NHPVA No. (%)** | **HPVA No. (%)** | ***P* value^a^** |
| --- | --- | --- | --- |
| **Age (years)** |  |  | **0.008** |
| <37 | 7(22.6%) | 24(77.4%) |  |
| ≥37 | 18(7.7%) | 215(92.3%) |  |
| **Figo stage** |  |  | **0.000** |
| I | 10(5.4%) | 176(94.6%) |  |
| II | 10(14.9%) | 57(85.1%) |  |
| III | 3(37.5%) | 5(62.5%) |  |
| IV | 2(66.7%) | 1(33.3) |  |
| **Tumor size(cm)** |  |  | **0.005** |
| <4.5 | 16(7.2%) | 205(92.8%) |  |
| ≥4.5 | 9(20.9%) | 34(79.1%) |  |
| **Differentiation** |  |  | 0.088 |
| Good | 3(27.3%) | 8(72.7%) |  |
| Moderate | 14(10.0%) | 126(90.0%) |  |
| Low | 8(7.1%) | 105(92.9%) |  |
| **Stromal invasion** |  |  | **0.038** |
| <1/3 | 4(6.0%) | 63(94.0%) |  |
| 1/3-2/3 | 4(4.9%) | 77(95.1%) |  |
| ≥2/3 | 17(14.7%) | 99(85.3%) |  |
| **Nerve invasion** |  |  | **0.001** |
| Negative | 18(7.6%) | 220(92.4%) |  |
| Positive | 7(26.9%) | 19(73.1%) |  |
| **LVI** |  |  | 0.310 |
| None (0) | 15(8.3%) | 165(91.7%) |  |
| Focal (1-4) | 7(13.2%) | 46(86.8%) |  |
| Moderate (5-9) | 3(16.7%) | 15(83.3%) |  |
| Extensive (≥10) | 0(0.0%) | 13(100.0%) |  |
| **LNM** |  |  | 0.096 |
| Negative | 16(7.8%) | 188(92.2%) |  |
| Positive | 9(15.0%) | 51(85.0%) |  |
| **Parametrium invasion** |  |  | 0.949 |
| Negative | 23(9.5%) | 219(90.5%) |  |
| Positive | 2(9.1%) | 20(90.0%) |  |
| **Surgical margin** |  |  | **0.001** |
| Negative | 19(7.8%) | 225(92.2%) |  |
| Positive | 6(30.0%) | 14(70.0%) |  |
| **HPV subtype** |  |  | **0.016** |
| HPV 16 | 3(4.5%) | 64(95.5%) |  |
| HPV 18 | 4(5.6%) | 68(94.4%) |  |
| Other subtypes | 0(0.0%) | 18(100.0%) |  |
| Not available | 0(0.0%) | 1(100.0%) |  |
| Negative | 18(17.0%) | 88(83.0%) |  |
| **MMR** |  |  | 0.933 |
| dMMR | 2(10.0%) | 18(90.0%) |  |
| pMMR | 23(9.4%) | 221(90.6%) |  |
| **P16** |  |  | **0.000** |
| Negative | 9(37.5%) | 15(62.5%) |  |
| Positive | 16(6.7%) | 224(93.3%) |  |
| **Ki67** |  |  | **0.018** |
| <12.5 | 11(16.9%) | 54(83.1%) |  |
| ≥12.5 | 14(7.0%) | 185(93.0%) |  |
| **TACC3** |  |  | **0.006** |
| <3.7 | 16(15.7%) | 86(84.3%) |  |
| ≥3.7 | 9(5.6%) | 153(94.4%) |  |

aChi-square test; HPVA, HPV-associated adenocarcinoma; NHPVA, nonHPV-associated adenocarcinoma; LVI: lymph vascular invasion; LNM, lymph node metastasis; MMR: mismatch repair; dMMR: deficient mismatch repair; pMMR: proficient mismatch repair; other subtypes: HPV45, HPV16/18, HPV18/45, HPV73/35/81, HPV53/56/66, HPV26/51/82, HPV18/39/59/68.

| **Supplementary Table 2. Diagnostic performances of studied testing for ECA patients.** | | | | | |
| --- | --- | --- | --- | --- | --- |
| **HPVA vs. NHPVA** | **AUC** | **95%CI** | **Sensitivity** | **Specificity** |  |
| **TACC3 IHC** | 0.640 | 0.526-0.754 | 0.640 | 0.640 |  |
| **P16 IHC** | 0.649 | 0.519-0.779 | 0.937 | 0.360 |  |
| **HPV subtype** | 0.675 | 0.567-0.783 | 0.630 | 0.720 |  |
| **TACC3+ HPV subtype** | 0.729 | 0.627-0.830 | 0.630 | 0.720 |  |

AUC, area under curve; CI, confidence interval; HPV subtypes were detected by PCR.
